# Cellular Senescence in Lung Fibrosis

**DOI:** 10.3390/ijms22137012

**Published:** 2021-06-29

**Authors:** Fernanda Hernandez-Gonzalez, Rosa Faner, Mauricio Rojas, Alvar Agustí, Manuel Serrano, Jacobo Sellarés

**Affiliations:** 1 Department of Pulmonology, ICR, Hospital Clinic, 08036 Barcelona, Spain; fhernandez@clinic.cat (F.H.-G.); aagusti@clinic.cat (A.A.); 2 Institute for Research in Biomedicine (IRB Barcelona), The Barcelona Institute of Science and Technology (BIST), 08028 Barcelona, Spain; manuel.serrano@irbbarcelona.org; 3 Instituto de Investigaciones Biomédicas August Pi i Sunyer (IDIBAPS), 08036 Barcelona, Spain; rfaner@clinic.cat; 4 Division of Pulmonary, Allergy and Critical Care Medicine, Department of Medicine, The Ohio State University Wexner Medical Center, Columbus, OH 43210, USA; Mauricio.Rojas@osumc.edu; 5 Centro de Investigación Biomédica en Red Enfermedades Respiratorias (CIBERES), 28029 Madrid, Spain; 6 Catalan Institution for Research and Advanced Studies (ICREA), 08010 Barcelona, Spain

**Keywords:** pulmonary fibrosis, senescence, senolytics, senomorphics, aging

## Abstract

Fibrosing interstitial lung diseases (ILDs) are chronic and ultimately fatal age-related lung diseases characterized by the progressive and irreversible accumulation of scar tissue in the lung parenchyma. Over the past years, significant progress has been made in our incomplete understanding of the pathobiology underlying fibrosing ILDs, in particular in relation to diverse age-related processes and cell perturbations that seem to lead to maladaptation to stress and susceptibility to lung fibrosis. Growing evidence suggests that a specific biological phenomenon known as cellular senescence plays an important role in the initiation and progression of pulmonary fibrosis. Cellular senescence is defined as a cell fate decision caused by the accumulation of unrepairable cellular damage and is characterized by an abundant pro-inflammatory and pro-fibrotic secretome. The senescence response has been widely recognized as a beneficial physiological mechanism during development and in tumour suppression. However, recent evidence strengthens the idea that it also drives degenerative processes such as lung fibrosis, most likely by promoting molecular and cellular changes in chronic fibrosing processes. Here, we review how cellular senescence may contribute to lung fibrosis pathobiology, and we highlight current and emerging therapeutic approaches to treat fibrosing ILDs by targeting cellular senescence.

## 1. Introduction

For several decades, understanding lung fibrosis as a process that limits lifespan has challenged scientists. Progressive loss of lung function due to pulmonary fibrosis contributes significantly to the ever-increasing burden of chronic disease throughout the world. Around half of deaths in the developed world are attributable to fibrotic diseases, including idiopathic pulmonary fibrosis (IPF), the most common fibrotic interstitial lung disease (ILD) characterized by progressive and irreversible respiratory failure and death [1,2]. The phenomenological complexity existing in the lung fibrosis process has led, over the years, to a rising number of hypotheses about the specific cellular and molecular causes. The most prominent feature of lung fibrosis is a gradual age-related loss of function that occurs at the molecular, cellular, and tissue levels. The lack of somatic maintenance and repair functions and the stochastic enforcement of damage may explain the marked variability of cellular mechanisms that appear to be involved in aging phenotypes, such as lung fibrosis [3,4].

Fibrosis and wound healing are essentially interwoven processes, driven by a cascade of injury, inflammation, fibroblast proliferation and migration, matrix deposition and remodelling. Pathological fibrogenesis that occurs in many diverse organs and diseases is a dynamic process involving complex interactions between epithelial cells, fibroblasts, immune cells (macrophages, T-cells), and/or endothelial injuries [5,6]. There are many extrinsic hazards known to induce injury to lung epithelium—infections, exposures to organic or inorganic components, cigarette smoking, and so forth—while there is also damage of unknown aetiology. As a response to lung injury, many interrelated wound-healing pathways are activated in order to facilitate the repair, turnover, and adaptation of lung tissue [7]. However, although their aetiology and causative mechanisms varies, the different fibrotic lung diseases all fail to properly eliminate inciting factors, leading to continued tissue damaging with an abnormal and exaggerated accumulation of extracellular matrix (ECM) components and collagen deposition. Another hallmark of lung fibrosis is that older individuals display impaired ability to restore tissue homeostasis, heal wounds and resolve fibrosis, resulting in tissue scarring and irreversible organ damage [8]. In this regard, emerging studies highlight that one of the most important underlying mechanisms implicated in the pathobiology of age-related diseases such as pulmonary fibrosis is cellular senescence.

Cellular senescence refers to the essentially irreversible transformation that cells experience upon unrepairable damage [9,10]. The two main transformations are: (1) inability to proliferate; and (2) abundant pro-fibrotic and pro-inflammatory secretome. The first property, inability to proliferate, is relevant for cells proliferating in vitro and for cancer cells and explains the potent tumour suppressive activity of cellular senescence. Indeed, the term senescence (from Latin, senex, a Latin word meaning a man of old age as a stock figure) was formally applied by Hayflick and colleagues to cells that had a limited ability to divide in culture (despite the presence of space, nutrients and growth factors in the medium), remaining viable for many weeks [11]. The irreversibility was considered a key factor for discerning cellular senescence from quiescence and transient cell cycle arrest, bringing senescence into the spotlight as a cancer-suppressor and a possible contributor to aging [9,12,13]. This observation was immediately linked with the hypothesis stemming from the fact that tissue regeneration impairs with age [10,14]. For many years, cellular senescence was considered a detrimental process as it contributes to impairment of tissue restoration and function. The number of senescent cells gradually increases with age, and the presence of senescent cells is a common finding in age-related pathologies. However, our understanding of the biology of senescence in an evolutionary context has led us to think about cellular senescence as an essential mechanism of antagonistic pleiotropy. This concept encompasses processes that are meant to be beneficial to the health of young organisms (as a strong tumour-suppressor mechanism, or integrating physiologically programmed mechanisms during development), but also can demonstrate deleterious effects in older organisms, most likely by promoting chronic inflammation and fibrosis that leads to both degenerative and hyper-plastic pathologies [15,16,17,18,19]. As discussed below, antagonistic pleiotropy is key to understanding many aspects of lung fibrosis, especially the relationship between aging, cellular senescence and lung fibrosis. In the lung, there is a relatively straightforward relationship to several environmental factors, so the setting in which cellular senescence develops is fraught with dangerous stressors, including DNA damage and telomere attrition, oncogenic signalling activation, epigenomic stress, redox imbalance, or mitochondrial biogenesis dysfunction [20,21]. This attribute might also explain the vulnerability of the lung to increases in senescence-inducing conditions that promote the loss of architectural integrity and elasticity, and subsequent pulmonary function impairment. Here, we review recent progress in understanding emerging insights into the mechanisms underlying cellular senescence and aging in lung fibrosis. We focus on the susceptibility of the lung to this process, and the detrimental role of cellular senescence in lung fibrosis pathobiology. Additionally, we discuss the critical altered mechanisms of the most implicated lung cell types whereby cellular senescence contributes to fibrotic lung diseases. Finally, we emphasize current and novel therapeutic approaches to treating fibrosing ILDs by targeting cellular senescence.

## 2. Perspective on Aging and Cellular Senescence in Lung Fibrosis

Over the past years, significant progress has been made in our understanding of the biology of lung fibrosis. The pathogenesis of fibrotic lung tissue remodelling is a dynamic orchestrated process and is thought to be strongly linked to aging [4,22]. For example, the incidence and prevalence of IPF appears to be remarkably higher in older ages; in fact, most IPF patients are older than 65 years at the time of diagnosis [23,24]. IPF is a progressive, irreversible and fatal lung disease characterized by scarring and thickening of the interstitial lung tissue leading to cough, dyspnoea and, ultimately, respiratory failure and death [2,25]. Although the prevalence of the disease has been found to be increasing overtime, it remains unclear whether this reflects better recognition or a real increase in incidence. For the moment, only limited therapeutic strategies exist to treat chronic lung fibrosis, and the two available drugs (Nintedanib and Pirfenidone) are only modestly effective at reducing the decline of lung function over a one-year follow-up [26,27]. Therefore, as life expectancy in developed countries is expected to increase in the coming decades, there is an urgent need to explore mechanisms for the pathogenesis of IPF and other fibrosing interstitial lung diseases, and develop novel therapeutics and interventions.

**Figure 1:**
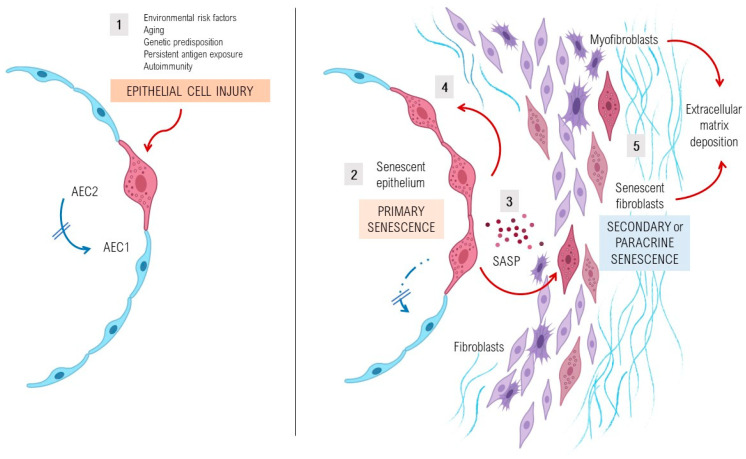
Pathogenesis and perpetuation of fibrosing interstitial lung diseases. Environmental risk factors, age-related cell perturbations, genetic predisposition, persistent exposure to antigens and autoimmune diseases are identified as important factors that increase susceptibility to epithelial lung injury acting as initial triggers of the disease. In an early phase, shown in the left part of the figure (1), epithelial type 2 cells (AEC2) show several markers of stress, activation, and senescence, as they fail to respond to stress resulting in an impaired capacity of the lung to regenerate. In a later phase, and especially after repeated alveolar damage, sustained aberrant activation and senescence of the epithelium (2) leads to the hyperactive secretion of high levels of pro-fibrotic growth factors, cytokines, chemokines and matrix metalloproteinases, collectively known as senescence-associated secretory phenotype (SASP) factors (3), that promote cellular senescence of the adjacent AEC2 (4). This initial event is encompassed in a process called primary senescence, which is thought to be characterized by the appearance of a senescent lung epithelium which persists by adapting its metabolic pathways and becoming resistant to apoptosis. In this later phase, a dysregulated crosstalk between the senescent epithelium and the mesenchymal cells through SASP factors will give rise to the accumulation of fibroblasts and myofibroblasts, resulting in an increased production and deposition of extracellular matrix components (5). A considerable part of fibroblasts and myofibroblasts will present a stressed and senescent phenotype, including resistance to apoptosis, which entails a process called secondary or paracrine senescence. As a consequence, in the context of senescence in lung injury and repair, the accumulation of extracellular matrix components in the interstitium of the lung will lead to lung fibrosis.

In this regard, a complex interplay of environmental and genetic factors, age-related processes and epigenetic reprogramming leads to profound age-related cellular alterations in alveolar epithelial cells (AECs), immune cells, and fibroblasts from fibrotic lungs [28] (Figure 1). Evolutionary theories of the pathogenesis of lung fibrosis propose all the “hallmarks of aging” as major mechanisms of disease, including genomic instability, telomere attrition, epigenetic alterations, loss of proteostasis, deregulated nutrient sensing, mitochondrial dysfunction, cellular senescence, stem cell exhaustion, and altered intercellular communication [14]. A growing body of evidence has provided a proof-of-concept that cellular senescence and the senescence-associated secretory phenotype (SASP) might be a key potential pathogenic phenotype, as they are associated with aging [29,30]. It has been shown that aged and diseased tissues of multiple multicellular organisms are more abundant in senescent cells [10,31,32]. Evidence provides support to the idea that senescent cells can fuel the hallmarks of a variety of aging phenotypes and overt age-related diseases, largely through the cell paracrine effects of the SASP [33]. Indeed, certain SASP components appear to act in a paracrine manner to reinforce the growth arrest of the exposed adjacent cells, which involves a process called secondary or paracrine senescence. Many of the cellular disruptions described in age-related processes have been shown to be present in epithelial and mesenchymal lung cells from patients with lung fibrosis [34]. As an example, gene mutations related to telomere maintenance and telomere attrition have been identified with DNA damage and a senescent phenotype in lung alveolar type 2 epithelial cells (AEC2s), inducing impairment in their regenerative capacity, and most of them have been demonstrated in patients with inherited IPF [35,36,37,38,39,40,41,42,43]. Animal models with telomere deficiency in telomerase reverse transcriptase (TERT) have been shown to develop lung fibrosis with higher frequency after repeated low doses of bleomycin [44]. Moreover, growing body of evidence suggests that aging induces mitochondrial impairment in different cells associating metabolic changes, and has been shown to induce cellular senescence, a process called mitochondrial dysfunction-associated senescence [45]. Interestingly, through the inhibition of malic enzymes (ME1 and ME2), p53 regulates cell metabolism and proliferation, and the downregulation of ME1 and ME2 also modulates the outcome of p53 activation, leading to the decisive induction of senescence, but not apoptosis, whereas enforced expression of either malic enzyme abolishes senescence [46].

Furthermore, recent efforts have led to the development of network theories of aging, in which the contribution of several mechanisms synergise and interact. For instance, one of the possible explanations of the key issue of the increasing number of senescent cells in the aged lung and their persistence at sites of age-related pathology is that functional and structural changes in the immune system, also termed immunosenescence, lead to a less efficient clearance of senescent cells facilitating the occurrence of lung fibrosis [10]. Nevertheless, although the manner in which all these mechanisms interrelate and promote cellular senescence in the pathobiology of lung fibrosis is still not clear, several initiatives to identify signalling pathways are increasingly evident and becoming more urgently required.

## 3. Lung Susceptibility to Cellular Senescence

Under normal conditions, AEC2s can self-renew and differentiate into type I alveolar epithelial cells and therefore are considered as alveolar progenitor cells [47,48]. However, in age-related diseases such as lung fibrosis, a decrease of the renewal capacity and disfunction of distinct lung’s cell types, as well as AEC2s or lung fibroblasts, is a widely known phenomenon. Cellular senescence of AEC2s and lung fibroblasts of patients with IPF determine a cellular phenotype with the hyperactive secretion of a set of pro-fibrotic SASP’s cytokines, growth factors, angiostatic and procoagulant mediators, and proteases that promote an abnormal wound healing response [20,49,50]. Activated AEC2s express several mediators (platelet-derived growth factor, transforming growth factor β1 [TGFβ1], tumour necrosis factor, osteopontin and CXC chemokine ligand 12, and endothelin-1), promoting profibrotic responses and inducing the migration of mesenchymal cells of different origins such as fibrocytes and resident fibroblasts [51]. Stress-induced senescent fibroblasts and myofibroblasts in the IPF lung secrete excessive amounts of ECM components, mainly fibrillar collagens and fibronectin, and are resistant to apoptosis, thus promoting the development of irreversible damage and lung fibrosis [49].

Repair and homeostasis of the lung occurs not only in an environment with increased oxidative stress but also in one that is highly susceptible to diverse exogenous hazards such as tobacco smoke, air pollutants, environmental antigens or infections, making cellular senescence extremely likely [20,52]. Mild oxidative stress as exerted by long-term hyperoxic conditions shortens substantially and irreversibly the proliferative life span of fibroblast, possibly by telomere shortening and DNA damage-mediated cell cycle arrest, establishing an interrelation with cellular senescence [53]. Moreover, changes in proteostasis at different levels in IPF lungs have been also associated with stress-induced senescence [54]. Inhibition of the proteasome by impaired autophagy, endoplasmic reticulum (ER) stress, misfolding protein response, and altered proteasome activity can induce a senescence phenotype in IPF lungs, increasing the pathology disturbing cellular reorganization and remodelling required for tissue repair [55,56,57,58]. Similarly, markers of ER stress in sporadic IPF lungs have been implicated in the stress-induced senescence by activating p21-dependant pathways, although the cause of ER stress in these patients is not well understood [59,60]. Mora, et al. elegantly showed that some potential mechanisms involve in inducement of ER stress, such as herpes virus infection or exposure to cigarette smoke, influencing susceptibility to age-related lung diseases [61,62]. However, it is possible that additional factors are implicated in this relationship between ER stress and stress-induced senescence in lung fibrosis.

## 4. Pathological Impact of SASP in Fibrotic Lungs

Alterations in the secretome of senescent cells with a chronic increment of production of several inflammatory secretory proteins, including plasminogen activator inhibitor-1 (PAI-1), matrix remodelling proteins (MMPs), growth factors such as TGFβ1, as well as other initial inflammatory stimuli, including IL-6, IL-8 and IL-1α, are present in IPF lungs. The SASP of these cells also has the potential to induce ER stress and enhances senescence through autocrine activity, but also can promote adjacent healthy cells to undergo senescence through paracrine senescence. In IPF lungs, the SASP effect of senescent AEC2s is detrimental as it is likely to induce a myriad of profibrotic mediators that drive pathological repair responses. SASP also consist of extracellular vesicles which are major mediators of intercellular communication through proteins, mRNAs, microRNAs, or organelles such as mitochondria. Novel studies suggest that NF-κB and CEBPB (CCAAT/enhancer-binding protein-β) are essential for the inducing of SASP factors, specially by inducing an autocrine feedback through IL-6 and IL-8 [63]. Moreover, stress-inducible kinase p38 mitogen-activated protein kinase and mTOR regulate SASP by altering NF-κB activity [64,65,66]. Additionally, a recent report indicates that senescent fibroblasts are an underlying driver of pulmonary fibrosis due to the secretion of leukotrienes (LT) as part of their SASP or the inducing of LT expression in other cells via a paracrine effect [50]. The profibrotic effects of the cystenyl LT-enriched SASP (especially LTC4 and LTD4) combined with the expression of CysTL1/L2 on the surface of the responding cells may be associated with the regulation of collagen expression by fibroblasts [67,68]. Therefore, an altered senescence response that promotes fibrosis via LT release but does not resolve because of the inability of senescent IPF fibroblasts to express COX2 and secrete antifibrotic prostaglandins (PGs) might be responsible for IPF progression. The incapacity of IPF senescent fibroblasts to secrete PGs could partly explain why the maintenance of senescent cells in the lungs is associated with increased fibrosis and consequently the progression of the disease. In this regard, it is important to better understand these dynamic and temporal changes in the SASP, as the role of the senescent cells may change during the progression of the disease [50]. There is increasing evidence that paracrine effects of the molecular pathways that regulate SASP can be manipulated and several experimental drugs are being tested, however, most studies are now at early preclinical phases.

## 5. Aged Fibroblasts and Extracellular Matrix Changes

Restoration to intact tissue after lung injury is crucial for proper lung homeostasis and function, and senescent cell accumulation also occurs during this process. However, through both in vitro and in vivo experiments, a mechanistic contribution of senescent cells to fibrotic lung disease has been recently been demonstrated [69]. It has been shown that the senescent phenotype of IPF lung fibroblasts is associated with increased levels of senescent markers (p16Ink4a and p21) and positive β-gal staining. While p21 expression is driven by telomere shortening-induced p53 activity, p16Ink4a is upregulated by telomere-independent unknown mechanisms [70]. Activating caspase-8 moiety in the Ink-Attac (INK-linked apoptosis through targeted activation of caspase) suicide gene product, which is expressed only in p16-positive senescent cells, significantly improved health and lifespan after initiation of the combined therapy with dasatinib plus quercetin by promoting the clearance of senescent cells [71]. Navitoclax (ABT-263, a specific inhibitor of the anti-apoptotic proteins BCL-2 and BCL-xL), effectively cleared senescent cells in young p16-3MR exposed to sublethal doses of radiation that induced a significant increase in senescent cells [72]. Therefore, improvements in lifespan after clearance of senescent cells suggests that persistent cellular senescence may contribute to several age-related diseases, including pulmonary fibrosis. It also has been shown that in normally proliferating cells, such as human fibroblasts, there is a dynamic feedback loop in which the long-term activation of the checkpoint gene *CDKN1A* (p21) induces mitochondrial dysfunction and reactive oxygen species (ROS) production, which is considered necessary for the establishment of the senescent phenotype [73]. In fibroblasts from IPF patients, this process particularly affects redox balance, controlled by NADPH oxidase 4 (NOX4) which has a crucial role in DNA damage, and is the key mediator of cellular antioxidant response NEF2-related factor 2. High expression of the NOX4 enzyme induces senescence in myofibroblasts by histone modifications and interferes with redox balance. It reduces reactive oxygen species, produces apoptosis of senescent myofibroblasts, and reverses established lung fibrosis in mice [8]. 

## 6. Loss of Epithelial Integrity

It has long been recognized that patients with sporadic or familial IPF present a dysfunctional alveolar epithelium associated with mutations and stress-induced senescence, which has a pivotal role in the aberrant lung injury-remodelling process. AEC2s in the IPF lungs express markers of senescence and apoptosis, which may indicate that the replacement of these cells might be a useful approach to attempting tissue repair and regeneration. It has been also proposed that stressed AEC2s in patients with lung fibrosis undergo an ineffective regeneration of the lung, so another pool of progenitor cells (KRT5+ Δp63+ cells) with persistent activation of the Notch signalling pathway mediates the repairment promoting micro-honeycombing rather than the regeneration of the normal alveoli epithelium [74]. Single cell RNA sequencing studies have shown an enhancement of senescent AEC2 cells in lung tissue from IPF patients compared to normal human lung tissue, which may activate profibrotic myofibroblasts by multiple canonical pathways, including TGFβ1 [75]. Likewise, signals from inflammatory cells and fibroblasts may activate epithelial cell gene expression in IPF. IPF cells express genes associated with the activation of TGFβ, HIPPO/YAP, p53, AKT-phosphoinositide-3-kinase (PI3K), and WNT signalling cascades consistent with extensive crosstalk among these pathways that likely functions in an integrated network. This abnormal cell signalling signature was most strongly expressed in the basal IPF cells, perhaps indicating their importance in the pathogenesis of pulmonary fibrosis [76]. Thus, the notion of a heterogeneity of transcriptional states of individual IPF cells challenges the concept of precise epithelial cell identities and supports the idea that strategies for the treatment of fibrosing lung diseases may need pharmaceuticals targeting multiple molecular pathways [29]. Similar to senescent fibroblasts, mitochondria dysfunction-associated senescence has also been described in AECs from IPF lungs. This mechanism driving aging is present in senescent AECs, leading to the accumulation of dysfunctional and dysmorphic mitochondria with altered fusion–fission dynamics and insufficient mitophagy, which induces an increase in oxidative stress and upregulation of markers of ER stress [77]. At the same time, and related to the timely repair of lung epithelia during pulmonary injury and repair processes, macrophages play important roles, although it is not fully addressed how these cells crosstalk with epithelial cells in advancing age. Although several studies have linked macrophage M2 polarization and their interaction with AECs to the progression of pulmonary fibrosis, the role of the subtype of M2 macrophages in lung repair and fibrosis is poorly understood, as it is not entirely known whether these cells have several metabolic states that regulate their functions, or how these subtypes interact with tissue environments [78]. Some findings implicate changes in the transcriptomic identity of the alveolar macrophages with age-related changes in the lung epithelial microenvironment, including alterations in the air-liquid interface formed by the epithelial lining fluid, a reduced number and altered function of alveolar macrophages with an age-related diminished response to granulocyte macrophage colony-stimulating factor (GM-CSF) secreted mainly by AEC2s cells [79]. In addition, a recent work showed that macrophage inhibitory cytokine-1 (MIC-1) secreted from senescent AECs enhances M2 macrophage activation and induces fibroblast activation in bleomycin-induced lung fibrosis [80]. Other studies also demonstrated that senescent AEC2s, through insulin-like growth factor-1 receptor or PAI-1 signalling, are necessary for the progression of pulmonary fibrosis and serve as chronic stimuli for macrophage activation in lung fibrosis [81,82]. Finally, a recent study demonstrated that although alveolar macrophages are long-lived lung-resident cells, changes in their number and transcriptional identity with age-related processes are not cell autonomous, although they instead might be shaped by interaction with the alveolar microenvironment independent of signalling in circulation [79]. Collectively, these findings suggest that strategies targeting the aging lung epithelial microenvironment may be crucial for restoring the alveolar macrophage-AECs axis function in aging.

## 7. Stem Cell Dysfunction

Another consequence of cellular senescence is the exhaustion of AEC2, the alveolar stem cells, leading to reductions in the regenerative capacity of these cells in the context of fibrogenic injury [83]. Recent studies demonstrate that telomere dysfunction can reduce the clearance of senescent cells by impaired immune system in patients with short telomeres and IPF [84]. Furthermore, AEC2-specific telomere dysfunction sensitizes mice to bleomycin challenge, highlighting the importance of a proliferative response in these cells after challenge. Based on this, some potential treatment strategies to restore or improve telomerase activity have been proposed [85]. In addition, recent reports have demonstrated extrapulmonary changes in the bone marrow-derived mesenchymal stromal cells (B-MSCs) of IPF patients, which might drive the systemic consequences of the disease [86]. Senescent IPF B-MSCs have been shown to present significant differences in mitochondrial function and the accumulation of DNA damage resulting in alterations in critical cell functions when compared with age-matched controls. Moreover, these cells can induce senescence in normal-aged fibroblasts, suggesting a possible link between senescence B-MSCs and the late onset of disease [86]. In this regard, there are several ongoing clinical trials to evaluate the safety and efficacy of MSCs for the treatment of IPF patients with initial encouraging results about safety. However, many questions are rising about mechanisms of action and usefulness for IPF treatment [29,87].

## 8. Future Perspectives

Left untreated, lung fibrosis leads to early death in all patients with fibrosing ILD, and limited therapeutic options exist. In IPF patients, single-agent treatments have a moderate effect and are likely to only slow the progression of the disease, mainly because of the considerable overload of the components that integrate the aberrant repair process [88]. Furthermore, no serum biomarker or technique has been validated for monitoring disease progression or assessing the fibrosis-driven component present in the lung. Current research efforts may lead to the integration of the normal physiology of aging and cellular senescence with the pathogenesis of age-related chronic entities such as pulmonary fibrosis. In this regard, one of the ultimate goals in the era of geroscience is to develop therapeutic approaches to several age-related diseases. Strong evidence suggests that cellular senescence has a clear role in the pathogenesis of several lung conditions such as pulmonary fibrosis. Thinking about senotherapy for IPF patients is especially attractive, although we are still left with some questions that need to be addressed. Importantly, are the mechanisms that lead to SASP and the senescence of epithelial cells and fibroblast through antiapoptotic pathways an unequivocal target in lung fibrosis? The answer to this question is not straightforward with the current evidence. However, pulmonary fibrosis is the best potential starting point for proof-of-principle studies of candidate senolytic drugs, compounds that selectively target and eliminate senescent cells or SASP-protective agents.

Several preclinical studies and subsequent clinical trials are focused on senescent cell antiapoptotic pathways (BCL-2/BCL-xL, PI3K/AKT, p53/p21/serpins, dependence receptors/tyrosine kinases, hypoxia-inducible factor-1α pathways). Currently, senotherapy includes several senolytic agents found to be targeting diverse network nodes that have been identified as critical for protecting senescent cells from apoptosis (Table 1) [89,90]. To accelerate the translation into clinical applications, bioinformatics approaches initially identified compounds, both drugs and natural products, whose mechanisms of action are found in drug substances already in use for other indications in humans, many as anti-cancer agents. These included Dasatinib and Quercetin, two non-specific and cell-type selective senolytic drugs. Dasatinib, a drug that differs from other non-senolytic tyrosine kinase inhibitors (such as Imatinib), promotes apoptosis caused by dependence receptors, in part by activating diverse Src kinase family members, KIT, platelet-derived growth factor receptors, and ABL and ephrin receptors. Dasatinib, which has been approved for clinical use in the United States since 2006 for the treatment of chronic myelogenous leukaemia and Philadelphia chromosome-positive acute lymphoblastic leukaemia, also impairs the viability of senescent preadipocytes by reducing the expression of p21, PAI-1 and BCL-xL [91]. Quercetin is a naturally occurring flavonoid present in apple peels, and selectively reduces the viability of senescent endothelial cells by inhibiting PIK3, other serpins and kinases. Depending on the cell type, for example, mouse embryonic fibroblasts, neither Quercetin nor Dasatinib are senolytic on their own, whilst the combination of both is senolytic. Based on their known targets, a combination therapy with Dasatinib and Quercetin not only was proved to clear senescent cells in vivo in chronologically aged progeroid mice, decreasing irradiation-induced cardiac dysfunction, but also impaired senescence and SASP markers (IL-6, IL8, MCP-1, PAI-1 and GM-CSF) in isolated AEC2s from bleomycin mouse models, improving lung function, and diminishing lung fibrosis induced after 5 days of bleomycin treatment in the transgenic Ink-Attac mouse model [69,91,92]. However, when the combined treatment was tested in mice with established lung fibrosis after 2–4 weeks of bleomycin administration, the expression of inflammatory markers was diminished, but attenuation of lung fibrosis failed [69]. In the first in-human clinical trial of senolytics Dasatinib and Quercetin, physical dysfunction was significantly and clinically meaningful improved in a preliminary study in patients with IPF, evaluated by testing 6-min walk distance, 4-metre gait speed and chair-stand time. It was an open-label study of intermittent Dasatinib plus Quercetin (3 days/week over 3 weeks) in stable IPF patients (N = 14) [93]. Moreover, correlations were found between change in function and change in SASP-related pro-inflammatory cytokines, matrix-remodelling proteases and micro-RNAs. This pilot study supports for the first time the feasibility of further clinical trials in pulmonary fibrosis using senolytics, but also supports the evaluation of the combination of these two drugs in larger randomized, controlled trials.

Another candidate, Navitoclax (ABT-263), which is a specific inhibitor of the anti-apoptotic proteins BCL-2 and BCL-xL, was identified from an agent library as having substantial off-target apoptotic effects on non-senescent cell types such as platelets and immune cells, making them potentially ‘panolytic’, causing apoptosis or dysfunction in multiple cell types other than senescent cells depending on the dosage [110,111]. Interestingly, this drug is shown to have both senolytic and anti-fibrotic properties, which have potential in reversing age-related fibrotic diseases such as pulmonary fibrosis. Treatment with Navitoclax was able to kill senescent fibroblasts and senescent epithelial cells, and effectively suppressed the expression of SASP in vitro, also in young p16-3MR mice [72,97]. Navitoclax acts as a selective, secure, and useful anti-fibrotic agent for reversing organ fibrosis in vivo [112,113]. It was shown that the treatment of established irradiation-induced pulmonary fibrosis in C57BL/6 mice with ABT-263 reduced senescent cells and reversed the disease [114]. However, Navitoclax has been critically limited by its severe neutropenic and thrombocytopenic effects [115]. In addition, targeting BCL-2 family proteins is senolytic in a limited number of cell types, suggesting that testing the restricted effect on every cell type of interest should be considered during the senolytic drug development process [29,97].

Although rapalog Everolimus (an inhibitor of the mechanistic target of mTOR), seemed to worsen patient outcome in previous clinical trials in IPF, another clinical trial using Rapamycin, another mTOR inhibitor, is currently being tested in IPF patients in a double-blind placebo-controlled pilot study (NCT01462006). Rapamycin has been recently proposed as a novel therapeutic target with the potential to impact multiple IPF patho-mechanisms influencing ECM production, metabolism, autophagy and senescence [116].

A preclinical in vivo study in aging mice with genetic and pharmacological targeting of NOX4 treated with bleomycin showed a significant decrease in senescent fibroblast and collagen deposition, and also reversed established lung fibrosis [8]. Another study in a rodent disease model also demonstrated the attenuation of persistent fibrosis using a NOX4 inhibitor [117]. In this regard, GKT137831, a dual inhibitor of NOX1 and NOX4, has been shown to diminish ROS production and senescence markers in IPF fibroblasts, and also interferes with critical events that regulate p53-dependent apoptosis and replicative senescence, suggesting that this compound might have senolytic effects in lung fibrosis [98,99]. Based on these data, a clinical trial testing GKT137831 was initiated in order to explore any differential effects on IPF patients (NCT03865927). Moreover, a PAI1 inhibitor (TM5275) blocked fibrosis in mice with bleomycin-induced lung fibrosis and protected AEC2s from senescence by targeting the p53 pathway [109].

Many other pharmaceutical drugs already used in humans have been shown to target senescence in animal models of lung fibrosis. These include antidiabetic drugs such as Metformin (prevents NF-κB nuclear translocation), cardiac glycosides (Digoxin), antihistamines (Rupatadine), other BCL-2 family protein inhibitors (ABT-737) and antibiotics (Azithromycin, Roxithromycin). These molecules target senescent cells by affecting pro-senescence or antiapoptotic proteins and controlling several SASP factors. However, new approaches to identifying and targeting these specific senescence-related pathways are under development.

## 9. Conclusions

Strong evidence supports a pathogenic role of cellular senescence in pulmonary fibrosis. Novel approaches have the potential to be a major milestone in our battle against fibrosing ILDs. Valuable pathogenic pathways have been recognized to have potential novel therapeutic targets that are likely to delineate the natural history of pulmonary fibrosis, and we expect that in the near future pioneering therapies targeting senescence will improve survival and quality of life in patients with this devastating disease.

Furthermore, the lack of reliable and measurable biomarkers for evaluating the effectiveness of senescence interventions in patients with pulmonary fibrosis also represents a serious challenge in this field. Perhaps the application of drug cocktails to affect several pathways in a simultaneous manner is the answer to overcome the multiple obstacles in the treatment of pulmonary fibrosis.

Implementing such approaches in routine clinical practice will be certainly a long-term process, but this study is a step in that direction.

## Figures and Tables

**Table 1:** Senescence-targeted treatments with emerging evidence in pulmonary fibrosis.

Agent	Mechanism of Action	Biological Activity	Reference
Dasatinib + Quercetin	↓ Bcl-xL, ↓ PI3K/Akt, ↓ p16, ↓ p21, ↑ cleaved caspase-3, ↑ apoptosis	↓ Senescent human cells in vitro; ↑ exercise capacity and ↓ SASP in mice; ↑ lifespan of progeroid and wildtype mice; ↓ physical dysfunction in human IPF patients	Zhu et al. [94], Fuhrmann-Stroissnigg et al. [95], Justice et al. [93], Xu et al. [96]
Navitoclax (ABT-263)	↓ Bcl-2	↓ Viability of senescent human lung fibroblasts, HUVECs, and murine embryonic fibroblasts	Zhu et al. [97], Chang et al. [72]
NOX1 and NOX2 dual inhibitor	↑ p53, ↑apoptosis	↓ ROS production and senescence markers in IPF fibroblasts	Eid et al. [98], Feng et al. [99]
Rapamycin	↓ mTOR, ↓ p16, ↓ p21, ↑ autophagy, ↓ ROS, ↓ NF-κB	↓ p16/p21-induced senescence in rodent and human cells in vitro	Demidenko et al. [100], Summer, et al. [101]
Cardiac glycosides (Digoxin)	↑ Apoptosis	↓ Senescent cells and fibrosis in a mouse model of lung fibrosis	Triana-Martinez, et al. [102]
ABT-737	↓ Bcl-X_L_, ↓ Bcl-W, ↑ apoptosis	↓ Senescent cells in lungs and epidermis in vivo	Yosef, et al. [103]
Azithromycin	↑ Autophagy	↓ Viability of human senescent lung and skin fibroblasts in vitro	Ozsvari, et al. [104]
Roxithromycin	↑ Autophagy	↓ Viability of human senescent lung and skin fibroblasts in vitro	Ozsvari, et al. [104], Zhang, et al. [105]
FOXO4-DRI peptide	↑ p53, ↑ apoptosis	↑ Apoptosis of senescent human lung fibroblasts in vitro	Baar et al. [106]
Metformin	↓ NF-κB, ↑ DICER1, ↓ Akt, ↓ p16, ↓ p21	↓ SASP in human senescent lung fibroblasts in vitro; ↓ senescent human lung fibroblasts in vitro	Moisseva et al. [107], Noren Hooten et al. [108]
PAI1 inhibitor (TM5275)	↑ p53, ↑ apoptosis	↓ fibrosis in mice with bleomycin mouse model; protected AEC2s from senescence	Disayabutr, et al. [109]

↓: Decrease; ↑: Increase; Bcl-xL: B-cell lymphoma extra-large; PI3K/Akt: Phosphoinositide-3-kinase/protein kinase B; SASP: Senescence-associated secretory phenotype; IPF: Idiopathic pulmonary fibrosis; Bcl-2: B-cell lymphoma 2; HUVECs: Human umbilical vein endothelial cells; NOX: NADPH oxidase; ROS: Reactive oxygen species; Bcl-W: B-cell lymphoma W; DICER1: Endoribonuclease Dicer 1; AEC2s: Alveolar type 2 epithelial cells.
